# Warming Effects on Periphyton Community and Abundance in Different Seasons Are Influenced by Nutrient State and Plant Type: A Shallow Lake Mesocosm Study

**DOI:** 10.3389/fpls.2020.00404

**Published:** 2020-04-09

**Authors:** Beibei Hao, Haoping Wu, Wei Zhen, Hyunbin Jo, Yanpeng Cai, Erik Jeppesen, Wei Li

**Affiliations:** ^1^Institute of Environmental and Ecological Engineering, Guangdong University of Technology, Guangzhou, China; ^2^Key Laboratory of Aquatic Botany and Watershed Ecology, Wuhan Botanical Garden, Chinese Academy of Sciences, Wuhan, China; ^3^Guangdong Provincial Key Laboratory of Water Quality Improvement and Ecological Restoration for Watersheds, Institute of Environmental and Ecological Engineering, Guangdong University of Technology, Guangzhou, China; ^4^Department of Bioscience, Aarhus University, Aarhus, Denmark; ^5^Wuhan Planning & Design Co., Ltd., Wuhan, China; ^6^Fisheries Science Institute, Chonnam National University, Yeosu, South Korea; ^7^Sino-Danish Centre for Education and Research, University of Chinese Academy of Sciences, Beijing, China; ^8^Limnology Laboratory, Department of Biological Sciences and Centre for Ecosystem Research and Implementation, Middle East Technical University, Ankara, Turkey

**Keywords:** climate warming, periphyton, seasonality, nutrient enrichment, structure complexity

## Abstract

Periphyton plays an important role in lake ecosystems processes, especially at low and intermediate nutrient levels where periphyton contribution to primary production can be similar to or exceed that of phytoplankton. Knowledge of how periphyton responds to key drivers such as climate change and nutrient enrichment is, therefore, crucial. We conducted a series of mesocosm experiments over four seasons to elucidate the responses of periphyton communities to nutrient (low and high, TN-0.33 mg L^–1^ TP-7.1 μg L^–1^ and TN-2.40 mg L^–1^ TP-165 μg L^–1^, respectively), temperature (ambient, IPCC A2 scenario and A2 + 50%) and plant type (two submerged macrophytes with different morphological structural complexity: *Potamogeton crispus* and *Elodea canadensis*, and their corresponding plastic imitations with similar size and structure). We found a noticeable seasonality in the abundance and composition of periphyton. In spring and summer, periphyton abundances were significantly higher in the turbid-high-nutrient state than in the clear-low-nutrient state, and in summer they were notably higher at ambient temperature than in climate scenario A2 and A2 + 50%. In contrast, periphyton abundances in autumn and winter were not influenced by nutrient and temperature, but they were notably higher on plants with a more complex morphological structure than simple ones. The genus composition of periphyton was significantly affected by nutrient–temperature interactions in all seasons and by plant type in winter. Moreover, periphyton functional composition exhibited noticeable seasonal change and responded strongly to nutrient enrichment and temperature rise in spring, summer, and autumn. Our results suggest that the effect of warming on periphyton abundance and composition in the different seasons varied with nutrient state and host plant type in these mesocosms, and similar results may likely be found under field conditions.

## Introduction

Periphyton plays an important functional role in lake nutrient cycles and food webs, especially at low and intermediate nutrient levels (Stevenson et al., 1996; McCormick and Stevenson, 1998; Vadeboncoeur and Steinman, 2002). The contribution of periphyton (on plants or lake bottom) to primary production in shallow lakes varies from as much as 99% to less than 1% along a eutrophication gradient (Vadeboncoeur and Steinman, 2002; Vadeboncoeur et al., 2003). Periphyton is sensitive and responds predictably and quickly to environmental changes, the latter due to a high turnover rate (McCormick and Stevenson, 1998; Schneider et al., 2012; DeNicola and Kelly, 2014). Multiple studies have shown periphyton to be a robust indicator of environmental state and thus useful in ecological assessment and management, providing higher-level understanding of the ecological status of shallow lake ecosystems (McCormick and Stevenson, 1998; Lavoie et al., 2004; Gaiser, 2009; Inglett et al., 2009; Danielson et al., 2012; La Hée and Gaiser, 2012; DeNicola and Kelly, 2014).

Nutrient and temperature are among the most important factors that drive biological processes and limit primary production of shallow lakes at a global scale (Falkowski et al., 1998; Behrenfeld et al., 2005; Elser et al., 2007). Periphyton growth in shallow lakes is often limited by nutrient availability (Hillebrand and Kahlert, 2001; Maberly et al., 2002; Lange et al., 2011; Hogan et al., 2014). It is well-established that nitrogen and phosphorus enrichment is tightly correlated with the increase in periphyton biomass and the change in periphyton community composition (Welch et al., 1992; Chetelat et al., 1999; Dodds et al., 2002; Wyatt et al., 2010; Gudmundsdottir et al., 2011). Climate warming is expected to affect the production and composition of both periphytic and planktonic algae through its direct effect on the physical properties of the water column (Smol et al., 2005; Jeppesen et al., 2009, 2011; Rühland et al., 2015) and indirectly by their effect on light availability and nutrient levels in lakes (Salmaso, 2005; Winder and Hunter, 2008; Rühland et al., 2015). How periphyton is affected by global warming is, however, debated (Mahdy et al., 2015). Some studies have shown that warming coupled with nutrient addition alters the temperature effects on periphyton, suggesting complex interactive effects of nutrient and temperature (Trochine et al., 2014; Piggott et al., 2015).

Habitat structure complexity, here expressed as the architecture and morphological characteristics of the host that provides attachment sites for periphyton, can influence the establishment and development of periphyton (Morin, 1986; Gosselain et al., 2005; Casartelli and Ferragut, 2018). Numerous studies have revealed that high habitat structure complexity leads to high periphyton biomass (Ferreiro et al., 2013; Pettit et al., 2016; Hao et al., 2017; Casartelli and Ferragut, 2018) and significantly affects the taxonomic composition of the periphyton attached to its surface (Blindow, 1987; Tunca et al., 2014; Hao et al., 2017). Hao et al. (2017) studied periphyton communities on natural and artificial macrophytes (plastic imitations of similar size and morphology as the real plants) with contrasting morphological structures during winter and found that although the periphyton composition differed significantly between the natural and artificial macrophytes, periphyton chlorophyll *a* (Chl*a*) was positively related to their structural complexity. However, it is an open question whether periphyton responds in a similar way to the structural complexity of natural/artificial macrophytes in other seasons. On the other hand, habitat complexity is a driving force determining consumer–resource interactions (Taniguchi et al., 2003), as higher habitat complexity hampers herbivore mobility and increases algal protection (Finke and Denno, 2002; Warfe and Barmuta, 2004), thereby reducing the grazing rates of herbivores on periphyton. Since seasonal temperature variation is associated with the dynamics of abiotic and biotic factors, including algal composition and herbivore–periphyton interactions, periphyton may respond differently to structural complexity in the different seasons (Rhee and Gotham, 1981; Hill et al., 2001).

We studied variations in the biomass development and composition of periphyton during four seasons in an existing mesocosm facility which has been running since 2003 (Liboriussen et al., 2005) having two nutrient levels (low and high) crossed with three temperatures (ambient, IPCC A2 scenario and A2 + 50%), and in our study supplemented with four plant types (two submerged macrophytes with different morphological structural complexity: *Potamogeton crispus* and *Elodea canadensis*, and their corresponding plastic imitations with similar size and structure). We hypothesized that the biomass and composition of periphyton would be strongly affected by increased temperature and nutrient enrichment in the growing seasons where the algal growth rate is high, whereas plant type would be most important in winter where environmental conditions are less favorable for growth.

## Materials and Methods

### Mesocosm Establishment

The mesocosm system we used is situated in Central Jutland, Denmark (56°140N, 9°310E) and includes 24 experimental tanks, which each is 1 m deep and has a diameter of 1.9 m (Supplementary Figure S1). The mesocosms are equipped with a flow-through system. Groundwater is added automatically every sixth hour and excess surface water removed by an overflow pipe (Supplementary Figure S1). They are designed to simulate simultaneously the warming effects on low nutrient (clear-state) and enriched (turbid-state) lakes, which have been in operation continuously since August 2003 (Liboriussen et al., 2005).

A factorial-design consisting of three temperature scenarios and two nutrient levels (six blocks in total) is applied to the mesocosms, each with four replicates. Among the 24 mesocosms, 8 are unheated (Ambient, AMB), while 8 are heated according to IPCC climate scenario A2 (warming, W) and 8 according to A2 + 50% (enhanced warming, EW). The temperature increase in the A2 scenario was calculated as the mean air temperature in one particular month with reference to period 1961 to 1990 and the temperature in the same month in 2017 to 2100 according to the IPCC climate model A2, the values being 2.74°C in May, 3.84°C in August, 3.76°C in November and 2.76°C in February (Liboriussen et al., 2005). The heating is controlled by electrical power, the temperature in AMB being used as reference.

Each temperature level is crossed with two nutrient levels. In this experiment, the mean concentrations of nutrient were TN-0.33 mg L^–1^ and TP-7.1 μg L^–1^ in the clear-state and TN-2.40 mg L^–1^ and TP-165 μg L^–1^ in the turbid-state. No additional nutrients are added to the clear-state mesocosms with low-nutrient levels, while nutrients are added to the turbid-state mesocosms to maintain a constant loading of 27.1 mg N m^–2^ day^–1^ and 7 mg P m^–2^ day^–1^. Nutrient addition was initiated in May 2003 when freshwater communities were established in the mesocosms, while heating was started in August 2003 when submerged macrophyte beds dominated in the non-enriched mesocosms and phytoplankton or filamentous algae dominated in the enriched mesocosms. Submerged macrophytes, mainly *Elodea canadensis* and *Potamogeton crispus*, appeared naturally in most of the clear mesocosms, whereas sparse shoots of these species emerged in only a few of the turbid mesocosms. More details of the experimental design and set-up are described in Liboriussen et al. (2005).

### Experimental Design

Our study was conducted in the mesocosms described above which had been running for 15 years when our experiment was conducted. Microorganisms in the mesocosms might have evolved and adapted to the current condition during this time period, which makes the experiment more realistic than if study was conducted in mesocosm that had just been established as is often the case. Our experiment was designed to test the responses of periphyton to nutrient, temperature and substrate type. Four types of substrate were selected for periphyton cultivation: the submerged macrophytes *P. crispus* and *E. canadensis* (hereafter termed natural *P. crispus* and natural *E. canadensis*) and their corresponding plastic imitations with similar size and structure (hereafter termed artificial *P. crispus* and artificial *E. canadensis*). *P. crispus* and *E. canadensis* have a widely different life-history and morphological structure. The aquatic monocot plant *P. crispus* has a branching stem, strip leaves (length 1.9 cm, breadth 0.6 cm), which germinates in winter and flowers in summer. *E. canadensis* has thin branched stems, leaves (length 3–8 cm, breadth 0.6–1 cm) in whorls of 3 without a petiole, and it grows faster in summer and flows in autumn. All the plant material was collected from the mesocosms. The artificial macrophytes (the plastic imitations) had a similar morphology to that of the natural plants and were obtained from a commercial vendor (Hengtong Aquarium Company, China).

**FIGURE 1:**
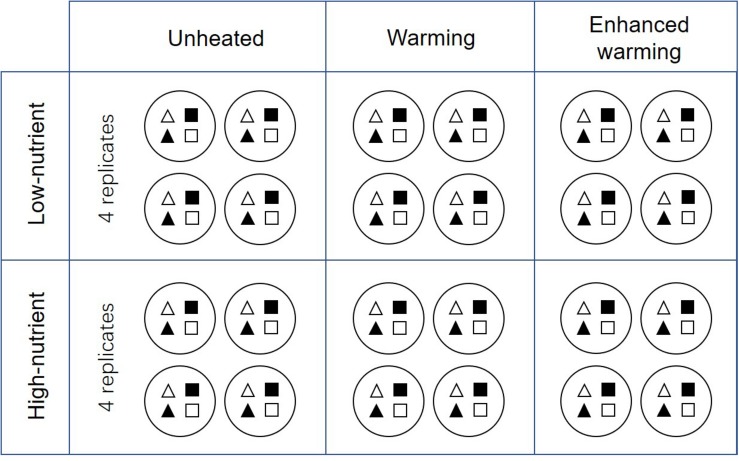
Schematic representations of the detailed experimental design. White-triangles represent natural *P. crispus*; black-triangles represent artificial *P. crispus*; white-quadrilaterals represent natural *E. canadensis*; black- quadrilaterals represent artificial *E. canadensis*. Note that the plots are grouped according to nutrient and temperature, and four plant types are listed regularly in the figure for illustrative purposes. In reality, the treatments were randomly assigned to the plots and four plant types were randomly assigned to the mesocosms.

Four identical experiments were conducted in May 2017, August 2017, November 2017, and February 2018, representing the four seasons (spring, summer, autumn, and winter) in Denmark. A 3-week experiment of periphyton cultivation was carried out independently in each season. In the beginning of each experiment, four types of plant substrate — natural *P. crispus* (N-PC), artificial *P. crispus* (A-PC), natural *E. canadensis* (N-EC) and artificial *E. canadensis* (A-EC) — were carefully rinsed to remove epiphytic algae from the surface. One individual of each plant substrate was randomly cultivated in a plastic barrel (height 29 cm, diameter 23 cm) filled with sediment (Figure 1). After that, one plastic barrel was hung in the center of each mesocosm at a depth of 25 cm below the water surface. Around the edge of the barrel, plastic netting (8 mm × 8 mm) was fixed to protect plants and periphyton from snail feeding. In total, 24 plastic barrels each cultivating four different plant substrates were hung in the 24 mesocosms (Figure 1). The 24 mesocosms represent six treatments: ambient temperature without nutrient addition (CON), warming without nutrient addition (W), enhanced warming without nutrient addition (EW), ambient temperature with nutrient addition (NP), warming with nutrient addition (W&NP) and enhanced warming with nutrient addition (EW&NP), each in four replicates to increase statistical power.

After 3 weeks’ cultivation, the four types of plant substrate were harvested from each tank. All above-ground natural/artificial plants were cut off and taken back to the laboratory. Here, the periphyton and zooplankton (Cladocera, Rotifera, and Copepoda) attached to each substrate were rinsed off carefully and extracted in 1000 ml water. Once rinsed, the plant substrates were placed in a plastic bag and digital photographs were taken. Photographs were scanned in ImageJ software and their surface area were estimated to twice that of the scanned area (Levi et al., 2015). The wash-off water was separated into two subsamples, one for measuring periphyton Chl*a* and the other for identification and enumeration of algae and zooplankton using an inverted microscope (Utermöhl, 1958). All the samples were identified in the same way and picophytoplankton was not of quantitative importance in any of the treatments. A 100∼200 ml subsample was filtered through a Whatman GF/C filter and photosynthetic pigments were extracted in 95% ethanol after which the solution was measured using a spectrophotometer (APHA, 1989). Periphyton Chl*a* was expressed as microgram Chl*a* per square centimeter of plant surface (μg cm^–2^), and the densities of periphyton and epiphytic zooplankton were expressed as the number of individuals per square centimeter of plant surface (ind cm^–2^). After enumeration by direct microscopic counting, periphyton taxa were classified into eight phyla and the density of each phylum was calculated. The relative abundances of three dominant phyla (Bacillariophyta, Chlorophyta, Cyanophyta) and others (periphyton taxa belonging to other five phyla) were calculated as the ratio (%) of each phylum density to total periphyton density.

Environmental variables were measured *in situ* in all mesocosms before each sampling event, including water temperature, dissolved oxygen (DO), pH and conductivity. In the laboratory, total nitrogen (TN) and total phosphorus (TP) were determined via colorimetry using a UV-visible spectrophotometer (UV-1800, Shimadzu, Germany). The surficial water in each mesocosm was collected for phytoplankton Chl*a* measurement, and the phytoplankton Chl*a* content was expressed as microgram Chl*a* per liter of water (μg l^–1^).

### Statistical Analysis

Three-way ANOVA and *post hoc* test were used to test the effects of nutrient, temperature, substrate type and their interactions on periphyton Chl*a*, density, the relative abundance of the main phyla and the diversity indexes of periphyton. In the three-way ANOVA design, the fixed factors included nutrient with two levels, temperature with three levels, nutrient^∗^temperature (treatment) with six levels and plant type nested within treatment with four levels. Two-way ANOVA (with nutrient, temperature and their interaction as fixed factors) were used to test the effects of nutrient, temperature and their interactions on environmental variables in each mesocosm. ANOVA was conducted in SPSS (IBM SPSS Statistic 20).

All the periphyton density data were square-root transformed and zooplankton data (attached to substrates) were 0–1 transformed to reduce the influence of a few dominant taxa, and a Bray–Curtis matrix of similarity among treatments was constructed. Two-way nested Analysis of similarities (ANOSIM, with treatment, plant type nested within treatment as the fixed factors) was conducted to compare the responses of periphyton and epiphytic zooplankton composition to treatment and plant type. To visually present possible differences, non-metric multidimensional scaling (NMDS) was performed using the R (version 3.6.3) vegan package. Besides, in order to expediently describe the seasonal variability in periphyton community structure, periphyton genera found in each season were classified into functional groups according to Reynolds et al. (2002) and Padisák et al. (2009). They classify all algae into functional groups according to their morphological, physiological and ecological characteristics. Two-way Permutational multivariate analysis of variance (PERMANOVA, with nutrient, temperature and nutrient^∗^temperature as fixed factors) were conducted to compare the responses of periphyton functional group composition to nutrient and temperature. ANOSIM and PERMANOVA were conducted in Primer (Version 5).

The relationship between environmental variables and periphyton genus composition in each season was analyzed in R (version 3.6.3) vegan package. For all periphyton data, the largest gradient length of detrended correspondence analysis was less than 3 standard deviation units. Redundancy analysis (RDA) was therefore considered most appropriate. RDA was carried out seasonally in the clear-low-nutrient state and the turbid-high-nutrient state using periphyton density data as response variable and environmental data as explanatory variables. Periphyton density data were Hellinger-transformed and all the environmental data were lg(*x* + 1) transformed to reduce variance. Environmental variables were selected using forward-selection procedure (*P* < 0.1) and permutation tests (with 999 permutations) were employed to test the significances of RDA models. RDA and permutation tests were conducted using the R (version 3.6.3) vegan package.

## Results

### Environmental Variables

During the experiment, the water temperature differed significantly among the three temperature levels in each season (Supplementary Table S1), and mean water temperatures were all lower than 20°C except in the A2 and A2 + 50% scenarios of the summer experiment (20.5°C and 22.5°C, respectively). Environmental variables such as phytoplankton chlorophyll *a* (Chl*a*), TN, TP, conductivity and pH did not vary significantly among the three temperature levels (Supplementary Table S1) but responded differently to nutrient in each season. The concentrations of phytoplankton Chl*a*, TN and TP in the four seasons, conductivity in August (summer) and pH in August (summer), November (autumn), and February (winter) were significantly higher in the turbid-high-nutrient state than in the clear-low-nutrient state. Besides, the concentration of dissolved oxygen (DO) decreased as the temperature rose in August (summer), being significantly lower in the A2 + 50% scenario (9.1 mg L^–1^) than in the A2 and ambient scenarios (11.4 mg L^–1^ and 13.0 mg L^–1^, respectively).

### Periphyton Abundance, Composition, and Diversity

#### Periphyton Chl*a* Among Treatments

**FIGURE 2:**
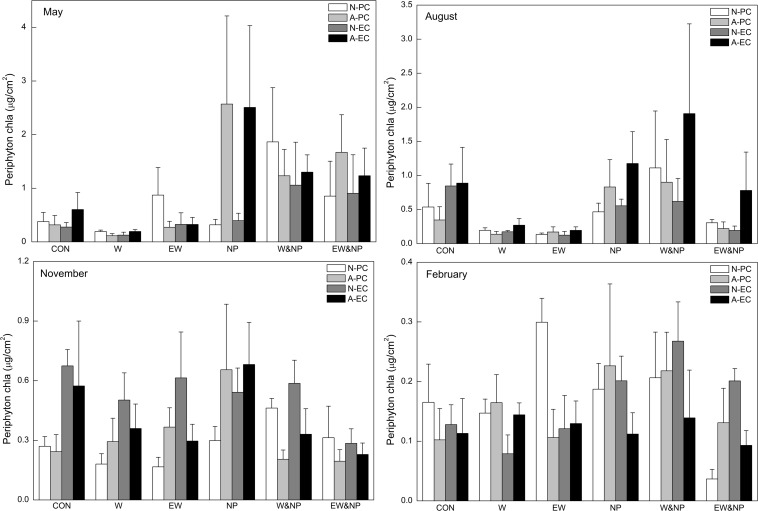
Periphyton Chl*a* on four types of plant substrate in each of six treatments divided into four sampling periods representing the four seasons. Plant substrates: natural *P. crispus* (N-PC), artificial *P. crispus* (A-PC), natural *E. canadensis* (N-EC) and artificial *E. canadensis* (A-EC); treatments: ambient temperature and without nutrient addition (CON), warming and without nutrient addition (W), enhanced warming and without nutrient addition (EW), ambient temperature and nutrient addition (NP), warming and nutrient addition (W&NP), enhanced warming and nutrient addition (EW&NP); sampling periods: May, August, November, and February (representing spring, summer, autumn, and winter).

In May and August, periphyton Chl*a* was significantly influenced by nutrient, and was thus significantly higher in the turbid-high-nutrient state than in the clear-low-nutrient state (Table 1 and Figure 2). A significant temperature effect on periphyton Chl*a* was only found in August where it was higher at ambient temperature than in the two warming scenarios (A2 and A2 + 50%) (Table 1 and Figure 2). Periphyton Chl*a* in November and February were significantly affected by plant type, being notably higher on natural *E. canadensis* than on the other plant types in November, and being statistically lower on artificial *P. crispus* than on artificial *E. canadensis* and natural *E. canadensis* in February (Figure 2).

**TABLE 1:** Results of three-way ANOVA comparing the effects of nutrient (two levels: low and high), temperature (three levels: ambient, warming, and enhanced warming), nutrient*temperature and plant type (four levels: natural ***P. crispus***, artificial ***P. crispus***, natural ***E. canadensis***,**** and artificial ***E. canadensis***) on chlorophyll *a* (Chl*a*) and density of periphyton.

	d.f.	May		August		November		February	
		*F*	*P*	*F*	*P*	*F*	*P*	*F*	*P*
**Chl*a* (μg/cm^2^)**									
Nutrient (N)	1	20.588	**0.000**	7.582	**0.007**	0.116	0.734	0.208	0.650
Temperature (T)	2	0.299	0.742	5.970	**0.004**	1.963	0.148	0.797	0.455
Nutrient*temperature	2	2.124	0.127	0.748	0.477	1.358	0.264	2.131	0.126
Nutrient*temperature (plant type)	18	0.664	0.834	0.418	0.980	2.110	**0.014**	1.855	**0.035**
**Density (ind/cm^2^)**									
Nutrient (N)	1	7.917	**0.006**	15.474	**0.000**	0.100	0.753	1.760	0.189
Temperature (T)	2	0.813	0.448	11.234	**0.000**	0.245	0.784	0.598	0.553
Nutrient*temperature	2	1.431	0.246	3.569	**0.033**	0.161	0.852	1.478	0.235
Nutrient*temperature (plant type)	18	0.889	0.593	1.510	0.112	1.843	**0.036**	4.015	**0.000**

*All bolded values in the tables are significant at p < 0.05.*

#### Periphyton Density Among Treatments

**FIGURE 3:**
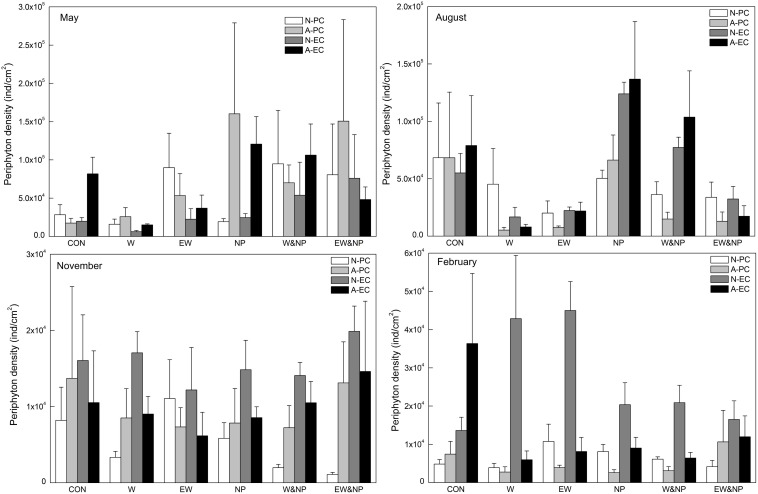
Periphyton density on four types of plant substrate in each treatment divided into season.

Periphyton density in May was strongly influenced by nutrient, being significantly higher in the turbid-high-nutrient state than in the clear-low-nutrient state (Table 1 and Figure 3). In August, periphyton density was significantly affected by nutrient, temperature and nutrient^∗^temperature, being statistically higher in the ambient temperature with nutrient addition treatment (NP) than in the warming without nutrient addition treatment (W) (Table 1 and Figure 3). In November and February, periphyton density was notably affected by plant type: natural *P. crispus*, artificial *P. crispus* < artificial *E. canadensis*, natural *E. canadensis* in November and artificial *P. crispus*, natural *P. crispus* < artificial *E. canadensis*, natural *E. canadensis* in February (Figure 3).

#### The Relative Abundance of Periphyton Among Treatments

In total, 78 periphyton genera (20 Bacillariophyta, 39 Chlorophyta, 8 Cyanophyta, and 11 others) were observed. Bacillariophyta were the dominant phyla and accounted for more than 60% of the abundance in each season (Supplementary Figure S2). *Achnanthes*, *Fragilaria*, and *Cocconeis* were the dominant genera, constituting different proportions in each season. The relative abundance of Cyanophyta in May was significantly influenced by nutrient and temperature: clear-low-nutrient state > turbid-high-nutrient state, ambient temperature < warming temperature (A2) (Supplementary Table S2 and Figure S2). In August, the relative abundances of Bacillariophyta, Chlorophyta, and Cyanophyta were significantly affected by plant type: artificial *E. canadensis* > other three plant types for Bacillariophyta, artificial *E. canadensis* < natural *P. crispus* and natural *E. canadensis* for Chlorophyta and artificial *E. canadensis* < artificial *P. crispus* for Cyanophyta, respectively (Supplementary Table S2 and Figure S2).

#### Periphyton Composition Among Treatments

**FIGURE 4:**
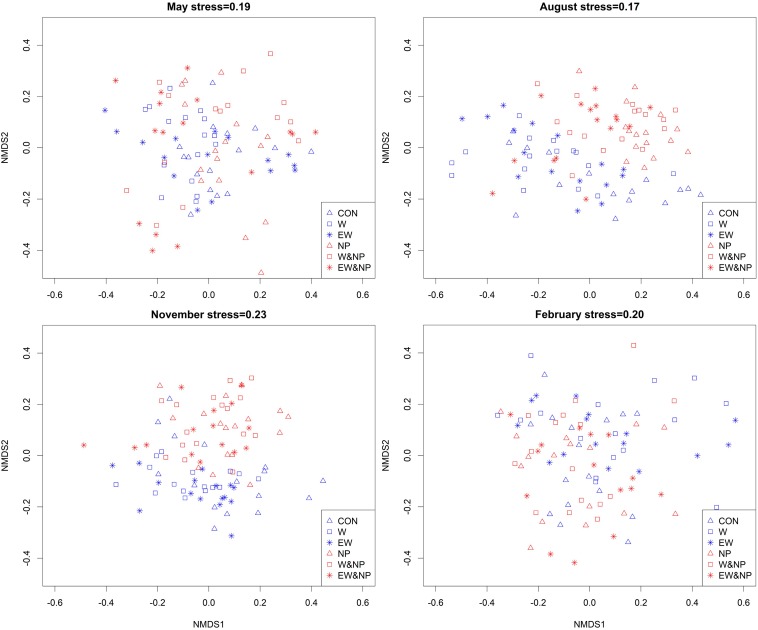
Non-metric multidimensional scaling (NMDS) plots of periphyton communities in six treatments based on Bray–Curtis similarities calculated from genus density (square-root transformed).

The genus composition of periphyton differed significantly among six treatments (Table 2). Results of *post hoc* pairwise tests showed that, excepting CON versus EW, NP versus W&NP and EW&NP in May, W versus EW in August, November, and February, and W&NP versus EW&NP in May, November and February, the periphyton composition differed significantly between any two treatments in all seasons (Figure 4 and Supplementary Table S3). (Treatment abbreviations are reiterated here for easy understanding. CON represents ambient temperature without nutrient addition; W represents warming without nutrient addition; EW represents enhanced warming without nutrient addition; NP represents ambient temperature with nutrient addition; W&NP represents warming with nutrient addition; EW&NP represents enhanced warming with nutrient addition). Significant plant type effects on periphyton composition were only found in February (Table 2), results of *post hoc* pairwise tests showing that the periphyton composition differed significantly between any two plant types (Supplementary Table S3).

**TABLE 2:** Results of two-way nested ANOSIM comparing the effects of treatment and plant type (nested in treatment) on the genus composition of periphyton.

	d.f.	May		August		November		February	
		Global R	*P*	Global R	*P*	Global R	*P*	Global R	*P*
Treatment	5	0.169	**0.007**	0.488	**0.001**	0.534	**0.001**	0.126	**0.030**
Treatment (plant type)	3	−0.077	0.958	−0.073	0.953	−0.044	0.811	0.296	**0.001**

#### Periphyton Diversity Among Treatments

The genus richness of periphyton was significantly higher at ambient temperature (mean value 18.53) than in the two warming scenarios (mean values 16.34 in warming scenario and 15.97 in the enhanced warming scenario, respectively) in August, while being significantly lower at warming temperature (mean value 12.28) than at enhanced warming temperature (mean value 15.03) and ambient temperature (mean value 15.19) in November. The Shannon–Wiener index of periphyton in May was notably lower in the turbid-high-nutrient state (mean value 2.35) than in the clear-low-nutrient state (mean value 2.48), while in August it was notably higher in the turbid-high-nutrient state (mean value 2.54) than in the clear-low-nutrient state (mean value 2.39). Significant plant type effects on the Shannon–Wiener index were found in May and February (Supplementary Table S4): artificial *E. canadensis* and *P. crispus* < natural *E. canadensis* and *P. crispus* in May and natural *E. canadensis* less than the other three types in February. The Shannon–wiener index in November was also significantly higher at enhanced warming (mean value 2.37) and ambient temperatures (mean value 2.33) than at warming temperature (mean value 2.09). In addition, the Pielou index of periphyton in May was significantly lower in the turbid-high-nutrient state (mean values 0.65) than in the clear-low-nutrient state (mean values 0.69), while it was significantly higher in the turbid-high-nutrient state (mean values 0.75) than in the clear-low-nutrient state (mean values 0.67) in August. The Pielou index in May, November and February was significantly affected by plant type (Supplementary Table S4): artificial *P. crispus* and *E. canadensis* < natural *P. crispus* in May, artificial *P. crispus* < natural *P. crispus* and artificial *E. canadensis* in November, and natural *E. canadensis* < the other three plant types in February.

### Periphyton Composition and Environmental Conditions

#### Relationship Between Periphyton Composition and Environmental Variables

**FIGURE 5:**
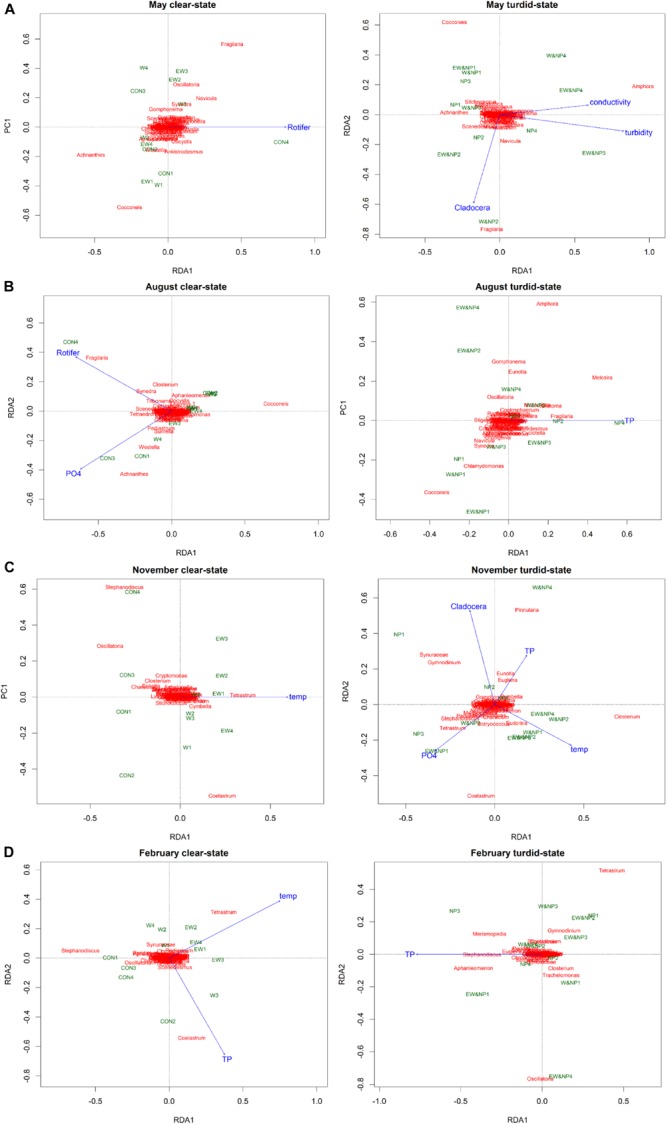
Tri-plots of RDA results showing the differences in periphyton genera, treatments and environmental variables between the clear-low-nutrient state and the turbid-high-nutrient state in **(A)** May, **(B)** August, **(C)** November, and **(D)** February.

Redundancy analysis revealed that the seasonal periphyton composition was significantly affected by different environmental variables in the clear-low-nutrient state and the turbid-high-nutrient state (Figure 5). In May, rotifer density was the only variable explaining 12% of variance (adjusted *R*^2^) of periphyton composition in the clear-low-nutrient state, while turbidity, Cladocera density and conductivity were the most significant variables, accounting for 37% of the variance (adjusted *R*^2^) in periphyton composition in the turbid-high-nutrient state (Figure 5A and Supplementary Table S5). In August, 23% of the periphyton in the clear-low-nutrient state was significantly affected and explained by rotifer density and PO_4_^3–^, while TP was the only variable explaining (7%) the variation in periphyton composition in the turbid-high-nutrient state (Figure 5B and Supplementary Table S5). In November, temperature was the only factor of significance in the clear-low-nutrient state, where it explained 8% of the variance in periphyton composition, while four environmental factors (temperature, ${\text{PO}}_{4}^{3-}$, Cladocera density and TP) were selected in the turbid-high-nutrient state and explained 43% of the periphyton (Figure 5C and Supplementary Table S5). In February, temperature and TP were the factors explaining 27% of the variance in periphyton composition in the clear-low-nutrient state, while TP was the only variable affecting periphyton composition, explaining 9% of the variance in the turbid-high-nutrient state (Figure 5D and Supplementary Table S5).

#### Functional Group Composition of Periphyton

During the four seasons, a total of 23 Reynolds functional groups were identified, differing in importance among seasons (Supplementary Figure S3). Functional group MP and T dominated in May and August, functional groups MP and L_O_ in November, and functional groups T and MP in February (Supplementary Figure S3). For the great majority of functional groups, such as MP, B, X2, P, J, F and H1, the periphyton density was generally higher in May and August than in November and February. For a few functional groups (such as T and D), the relative abundances increased in winter, coinciding with a decrease in functional group MP. Results of ANOSIM showed that the functional composition of periphyton was significantly influenced by nutrient, temperature, and nutrient^∗^temperature in May and August, by nutrient and temperature in November and by nutrient in February (Supplementary Table S6). Results of *post hoc* pairwise tests showed that, periphyton functional composition differed significantly between ambient and warming temperatures (A2 and A2 + 50%) in May, August and November, while no significant differences were found between the two warming scenarios (A2 and A2 + 50%) in all seasons (Supplementary Figure S3).

### Epiphytic Zooplankton

Apart from periphyton, 60 taxa of epiphytic zooplankton (12 Cladocera, 27 Rotifera, and 21 Copepoda) were also found in our study. The density of Cladocera in May was significantly higher in the turbid-high-nutrient state than in the clear-low-nutrient state, while the density of rotifers in August was significantly higher at ambient than at warming temperatures (A2 and A2 + 50%) (two-way ANOVA, *P* < 0.05). Correlation analysis revealed that the abundances of diatoms and green algae were positively correlated with rotifer abundance in May and August (Pearson correlation, *P* < 0.05). Besides, the composition of the epiphytic zooplankton community in the four seasons differed significantly among six treatments (two-way ANOSIM, *P* < 0.001 in four seasons), while no significant plant type effects on epiphytic zooplankton composition were found (two-way ANOSIM, *P* > 0.05).

## Discussion

### Differences in the Effects of Nutrient, Temperature, and Plant-Type on Periphyton Abundance

Our study showed that periphyton abundance (Chl*a* and density) responded differently to nutrient and temperature among the seasons. Nutrient enrichment significantly increased periphyton abundance in spring and summer (May and August) but had only a minor effect in autumn and winter (November and February). Rosemond et al. (2000) suggested that the effects of nutrient addition on periphyton biomass and productivity would be greater under high-light conditions, as the effects of nutrient and light on periphyton were highly interdependent. This may explain why the responses of periphyton abundance to nutrient addition were positive in spring/summer and neutral in autumn/winter.

A significant effect of temperature on periphyton abundance was only found in summer where Chl*a* and the density of periphyton were significantly reduced by warming (in both A2 and A2 + 50%). Higher temperature often leads to higher respiration than production due to the differential temperature scaling of photosynthesis and respiration (Allen et al., 2005; O’Connor, 2009; O’Connor et al., 2009) and, consequently, lower net production (Scharfenberger et al., 2019). Moreover, several studies have shown that warming increases the metabolism of herbivores and strengthens their top-down effects on periphyton (O’Connor, 2009; David, 2010; Gabriel et al., 2010; Carr and Bruno, 2013). These could be the possible reasons for the lower periphyton Chl*a* in the two warming scenarios in our study. In addition, Verbeek et al. (2018) revealed that nutrient availability altered the temperature effects on phytoplankton production, the effects being positive when resources were sufficient and negative when nutrient became scare. Supporting this view, periphyton Chl*a* and density were particularly reduced by warming in the clear-low-nutrient state in our summer experiment.

Plant-type had a significant effect on periphyton abundances in autumn and winter, periphyton Chl*a* and densities being significantly higher on natural/artificial *E. canadensis* than on natural/artificial *P. crispus*. It is to be expected that complex structure not only offers a larger surface area but also maximizes access to light for the growth of periphyton (Taniguchi et al., 2003; Tunca et al., 2014; Pettit et al., 2016; Hao et al., 2017). Besides, Tramonte et al. (2019) revealed that periphyton consumption by herbivores was greater in simplified than in complex habitats, which may explain the observed lower periphyton density on natural/artificial *P. crispus* whose habitat structural complexity was simple compared with natural/artificial *E. canadensis*. Periphyton abundance can also differ between a natural plant and the corresponding artificial plant as macrophytes may release nutrients (Carignan and Kalff, 1982; Hilt, 2006) and/or allelopathic compounds (Burkholder and Wetzel, 1990; Gross, 2003; Erhard and Gross, 2006) to promote and/or inhibit periphyton attachment. In our study, significant differences between natural and artificial *E. canadensis* were found in autumn and winter when *E. canadensis* was constantly decaying and released nutrients that potentially stimulated periphyton growth, perhaps explaining the higher periphyton Chl*a* on natural *E. canadensis* than on artificial *E. canadensis*. In sum, we found a complex response of periphyton abundance to nutrient addition, temperature increase and substrate type, varying with season and nutrient state. The results further indicate that periphyton abundance under warm-season and nutrient-poor conditions will be more vulnerable to short-term warming, whereas in the cool-season periphyton abundance will be more susceptible to the structural complexity of the substrate.

### Responses of Periphyton Composition to Treatments Differ Among Seasons

The ANOSIM results confirmed that periphyton composition was sensitive to the interaction between nutrient and temperature. Among seasons, the genus composition differed significantly between any two treatments except between W and EW, W&NP, and EW&NP. This suggests that the temperature difference between scenario A2 and A2 + 50% (mean value 1.60°C) was too low to affect the periphyton composition in both the clear-low-nutrient state and the turbid-high-nutrient state. Similar results have been reported by Larras et al. (2013) in a 2°C temperature increase study. Meanwhile, significant plant-type effects on periphyton composition were only found in winter characterized by a harsher environment. Consequently, resource availability becomes the main factor determining the community composition of periphyton, which can be easily influenced by submerged plants directly through the release of allelochemicals and/or nutrients (Erhard and Gross, 2006; Hilt, 2006) and indirectly by substrate structural complexity through the effects on light availability at plant surfaces (Taniguchi et al., 2003; Tunca et al., 2014). Under benign environmental conditions in other seasons, however, the periphyton community might be affected by interactions among the biotic/abiotic factors in shallow lakes (Mallory and Richardson, 2005; Correia et al., 2012), masking the substrate.

### Environmental Effects on Periphyton Composition

The RDA results revealed that temperature was only an important factor affecting the periphyton composition in autumn and winter (November and February) and that there was no significant difference between the clear-low-nutrient state and the turbid-high-nutrient state. Winter is normally associated with environmental minima, such as low temperatures associated with reduced solar radiation and low light intensity, the latter due to ice and snow cover, providing a harsh environment for primary producers (Gustina and Hoffmann, 2000; Marchand, 2014). In our study, the mean ambient temperatures in autumn and winter (3.9°C in November and 1.5°C in February) were low compared with spring and summer (16.1°C in May and 16.8°C in August), and most of the mesocosms were covered by ice and/or snow during the experiments in November and February. Menge and Sutherland (1987) argued that under stress conditions, such as those found in winter, biotic communities would be regulated by abiotic factors rather than by competition or predation. This may help explain why the periphyton communities were mainly controlled by physico-chemical factors, such as temperature and/or phosphorous, in the autumn and winter experiments of our study.

The RDA results for spring and summer revealed that the environmental variables explaining periphyton composition differed notably between the clear-low-nutrient state and the turbid-high-nutrient state. The RDA showed that periphyton composition could best be explained by rotifers in the clear-low-nutrient state and by turbidity or TP in the turbid-high-nutrient state. These results concur with those of previous studies demonstrating that the grazing effects of herbivores on periphyton are weakened by high nutrient availability (Osenberg and Mittelbach, 1996; Iannino et al., 2019). Grazers can change algal community composition through differential predation (Rosemond et al., 1993; Feminella and Hawkins, 1995) and herbivores prefer algae with upright, erect or filamentous morphologies over taxa with prostrate morphologies (Steinman et al., 1992; Rosemond et al., 1993). However, with nutrient enrichment, periphyton responds quickly while herbivores may not react immediately to the changes in food availability (Hansson, 1992); this time lag in the response of grazers to nutrient addition may result in failure of grazers to control algae composition. Besides, predation on grazers may also increase with warming (David, 2010; Jeppesen et al., 2010), leading to a weakening of the impact of herbivores on periphyton.

Multiple studies have reported that periphyton community composition is strongly influenced by seasonality (O’Reilly, 2006; Borduqui and Ferragut, 2012; De Souza et al., 2015). In our study, the functional group composition of periphyton also showed a noticeable seasonal change. The functional groups MP and L_O_ exhibited the highest densities and relative abundances in spring and summer, which can be linked to high light and high nutrient conditions (Bovo-Scomparin and Train, 2008; Yang et al., 2011). Functional group T, which is tolerant to light deficiencies (Reynolds et al., 2002), dominated the periphyton community in winter. Moreover, the periphyton community, which prefers warm water, exhibited higher relative abundances in spring/summer, such as functional groups B, J, and F (Jiang et al., 2013), whereas functional group D which prefers cold water (Jiang et al., 2013), showed higher proportions of periphyton in winter. Our results, therefore, support the view that functional group can be used as an approach to analyze the seasonal variability in algal community (Yang et al., 2011; Yu et al., 2012). Except for winter, the periphyton functional composition responded strongly to nutrient addition and temperature increase, which is consistent with the responses of periphyton genus composition to nutrient and temperature in spring, summer and winter. Similar results have been reported by Larson et al. (2012) who found that the functional and taxonomic composition of periphyton exhibited similar variation trends in response to environmental changes such as nutrient and light availability.

## Conclusion

We found a complex response of periphyton abundance to nutrient enrichment, temperature increase and the structure complexity of substrate, which varied with season. Periphyton abundance was strongly affected by nutrient addition and the temperature rise in spring and summer, whereas substrate structure was of great importance for periphyton abundance in autumn and winter when environmental conditions were harsh for periphyton growth. In contrast, the community composition of periphyton was significantly influenced by the interactions between nutrient and temperature and independent of seasonality. Our results suggest that the effect of warming on periphyton abundance and composition in the different seasons varied with nutrient state and host plant type in the shallow-lake mesocosms, and similar results are likely to occur in natural lakes.

## Data Availability Statement

The raw data supporting the conclusions of this article will be made available by the authors, to any qualified researcher with reasonable request.

## Ethics Statement

This study did not involve listed endangered or protected species. No specific permits were required for this study.

## Author Contributions

BH designed the study, performed the research, analyzed the data, and wrote the manuscript. HW performed the research, collected samples, and analyzed the data. WZ and HJ collected samples. YC, EJ, and WL contributed to the data analysis and revisions. All authors have reviewed, discussed and agreed to the authorship and submission of the manuscript for peer review.

## Conflict of Interest

WZ was employed by the company Wuhan Planning & Design Co., Ltd., Wuhan, China. The remaining authors declare that the research was conducted in the absence of any commercial or financial relationships that could be construed as a potential conflict of interest.

## Supplementary Material

FIGURE S1 Illustration and photo of the 24 flow-through experimental mesocosm setup studying how increased temperature will affect shallow lake systems (Liboriussen et al., 2005).

FIGURE S2 Relative abundances of Bachillariophyta (Bac), Chlorophyta (Chl), Cyanophyta (Cya) and others (Oth) in periphyton on four types of plant substrate in each treatment on a seasonal basis. CON represents ambient temperature without nutrient addition; W represents warming without nutrient addition; EW represents enhanced warming without nutrient addition; NP represents ambient temperature with nutrient addition; W&NP represents warming with nutrient addition; EW&NP represents enhanced warming with nutrient addition.

FIGURE S3 Functional group composition and density of periphyton in May (spring), August (summer), November (autumn) and February (winter).

TABLE S1 Results of two-way ANOVA comparing the effects of nutrient (two levels: low and high; d.f. = 1), temperature (three levels: ambient, warming and enhanced warming; d.f. = 2) and the interaction between nutrient and temperature (d.f. = 2) on environmental variables. All bolded values in the tables are significant at *p* < 0.05.

TABLE S2 Results of three-way ANOVA comparing the effects of nutrient (two levels: low and high), temperature (three levels: ambient, warming and enhanced warming), nutrient*temperature and plant type (four levels: natural *P. crispus*, artificial *P. crispus*, natural *E. canadensis* and artificial *E. canadensis*) on the relative abundance of Bacillariophyta, Chlorophyta and Cyanophyta in periphyton. All bolded values in the tables are significant at *p* < 0.05.

TABLE S3 Results of Pair-wise ANOSIM tests for treatment effects (nutrient*temperature) in May, August, November and February. CON represents ambient temperature without nutrient addition; W represents warming without nutrient addition; EW represents enhanced warming without nutrient addition; NP represents ambient temperature with nutrient addition; W&NP represents warming with nutrient addition; EW&NP represents enhanced warming with nutrient addition. All bolded values in the tables are significant at *p* < 0.05.

TABLE S4 Results of three-way ANOVA comparing the effects of nutrient (two levels: low and high), temperature (three levels: ambient, warming and enhanced warming), nutrient*temperature and plant type (four levels: natural *P. crispus*, artificial *P. crispus*, natural *E. canadensis* and artificial *E. canadensis*) on the Richness, Shannon-Wiener and Pielou indexes of periphyton. All bolded values in the tables are significant at *p* < 0.05.

TABLE S5 RDA results of environmental variables selected by forward-selection procedures and permutation tests for the RDA models in the clear-low-nutrient state and the turbid-high-nutrient state in May, August, November and February. P values represent the results of permutation tests for the models, and R2 and R2 adjusted are the cumulative proportions explained by the models. All bolded values in the tables are significant at *p* < 0.05.

TABLE S6 Results of two-way PERMANOVA comparing the effects of nutrient (two levels: low and high), temperature (three levels: ambient, warming and enhanced warming) and their interactions on the functional group composition of periphyton. All bolded values in the tables are significant at *p* < 0.05.
