# Supplementary figures and images for: Warming Effects on Periphyton Community and Abundance in Different Seasons Are Influenced by Nutrient State and Plant Type: A Shallow Lake Mesocosm Study
Source: Front Plant Sci. 2020 Apr 9;11:404. doi: 10.3389/fpls.2020.00404 (PMC7161416; doi:10.3389/fpls.2020.00404)

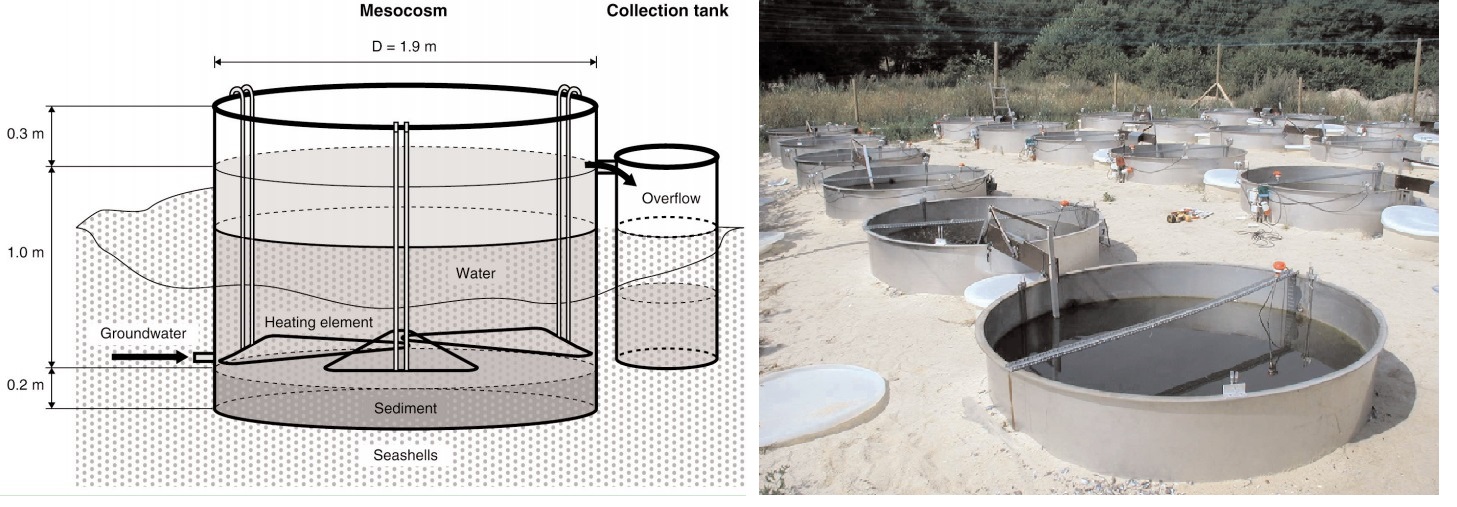

Supplement: FIGURE S1 — Illustration and photo of the 24 flow-through experimental mesocosm setup studying how increased temperature will affect shallow lake systems (Liboriussen et al., 2005). [file Image_1.jpg]

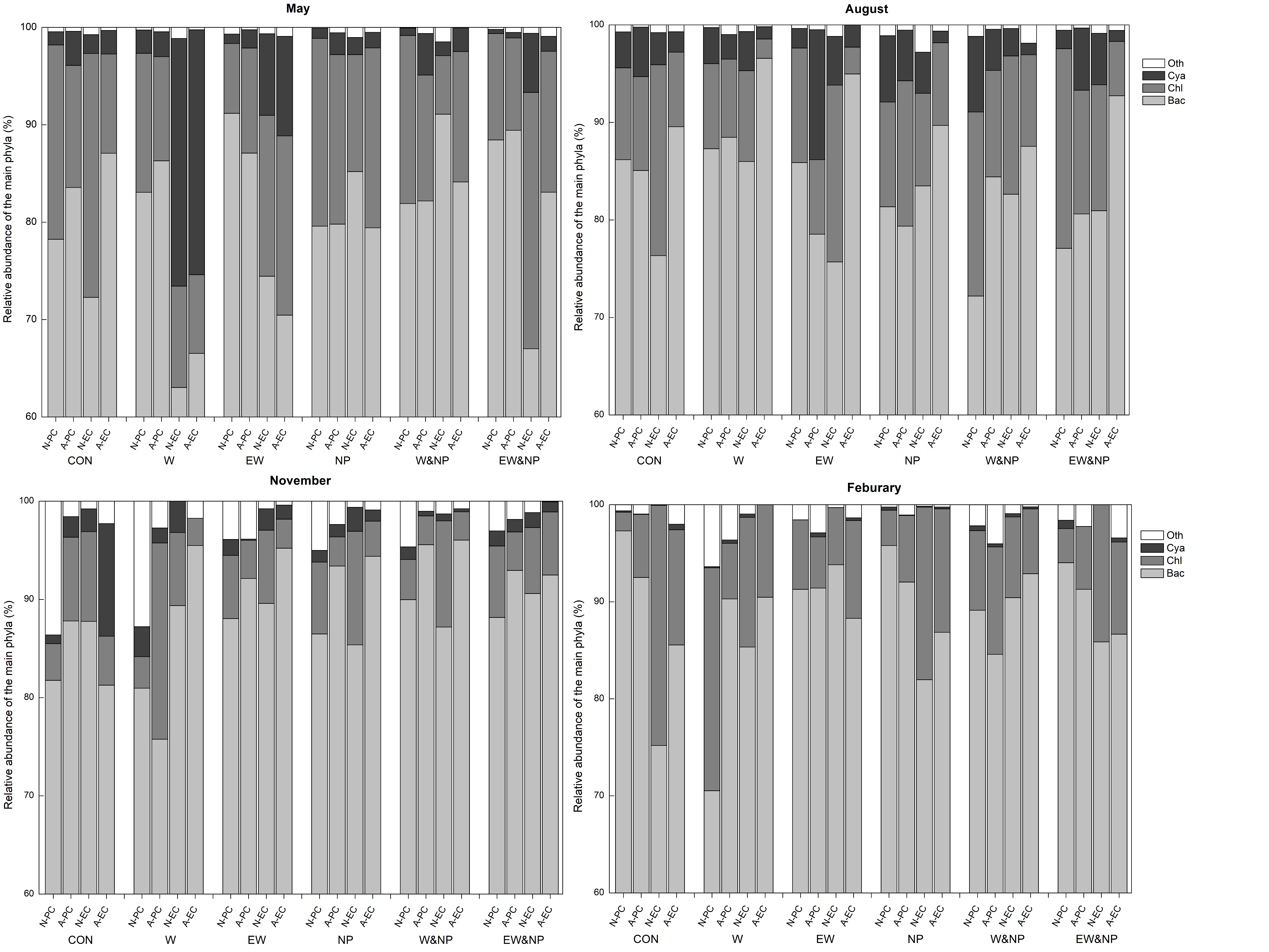

Supplement: FIGURE S2 — Relative abundances of Bachillariophyta (Bac), Chlorophyta (Chl), Cyanophyta (Cya) and others (Oth) in periphyton on four types of plant substrate in each treatment on a seasonal basis. CON represents ambient temperature without nutrient addition; W represents warming without nutrient addition; EW represents enhanced warming without nutrient addition; NP represents ambient temperature with nutrient addition; W&NP represents warming with nutrient addition; EW&NP represents enhanced warming with nutrient addition. [file Image_2.tif]
